# Genome Sequence of the Alcelaphine Gammaherpesvirus 1 Attenuated Laboratory Strain WC11

**DOI:** 10.1128/genomeA.01219-17

**Published:** 2017-11-09

**Authors:** Armin Ensser

**Affiliations:** Virologisches Institut, Universitätsklinikum Erlangen, Friedrich-Alexander-Universität Erlangen-Nürnberg, Erlangen, Germany

## Abstract

The complete genome sequence of the alcelaphine gammaherpesvirus 1 (AIHV-1) attenuated laboratory strain WC11 was determined from purified virion DNA. The viral light DNA (L-DNA) genome of 127,215 bp is mostly conserved compared to the pathogenic strain C500; however, 3.3 kb is deleted in two regions, affecting 4 of 10 AIHV-1-specific open reading frames.

## GENOME ANNOUNCEMENT

Originally assigned to the genus *Rhadinovirus*, alcelaphine gammaherpesvirus 1 (AIHV-1) and its closer relatives are now classified in the genus *Macavirus* (malignant catarrhal fever caused by the type species AIHV-1) in the subfamily *Gammaherpesvirinae*. Virion DNA was isolated from the supernatant of MDBK cells (ATCC CCL-22) infected with AIHV-1 attenuated strain WC11 (1) obtained from D. W. Verwoerd (Veterinary Research Institute, Onderstepoort, South Africa) as described previously (2). Infected tissue culture supernatant was precleared by centrifugation at 2,000 × *g* and WC11 virions were pelleted by ultracentrifugation at 50,000 × *g* with an SW28 rotor. The viral DNA of WC11 was further purified by CsCl density gradient centrifugation as described previously (3) and resuspended in 10 mM Tris-HCl and 1 mM EDTA (pH 8.0). Fifty nanograms of virion DNA was used for dual-indexed library preparation using the Nextera DNA sample preparation kit (Illumina) according to the manufacturer’s recommendations. Diluted libraries were paired-end sequenced (MiSeq reagent kit, 2 × 150 cycles and 2 × 300 cycles) with an Illumina MiSeq instrument. Reads were analyzed with CLC Genomics Workbench (Qiagen Bioinfomatics, Aarhus, Denmark) and trimmed (minimum length, 40 bp, quality, ≤0.05; ≤2 ambiguous nucleotides), and 586,262 reads were aligned (96.7% mapped, 89.5% as pairs; average total coverage, >579×; light DNA [L-DNA], >525; heavy DNA [H-DNA], >7,000) to the pathogenic strain C500 reference sequence (2).

Furthermore, four contigs were generated by *de novo* assembly (default options) and compared to strain C500 and searched against GenBank using BLASTn. The largest contig (110 kb; coverage, 536×) encompassed open reading frames (ORFs) A3 to 69.

Compared to the AIHV-1 reference genome (L-DNA [NCBI reference sequence NC_002531], H-DNA [GenBank accession no. AF005363 through AF005367]), the attenuated strain WC11 has the following deletions (positions relative to the C500 genome): left genome end at positions 1 to 1172, deleting viral microRNA (miR) cluster 1 (4) and most of ORF A1. The other region of viral miR cluster 2, which has no role in pathogenesis (4), is well conserved between WC11 and C500.

Wright et al. (5) and Handley et al. (6) observed attenuation-associated rearrangements of gene regions either next to or in terminal repeat elements at either end of the genome. A reproducible duplication in the C500 strain and bacmid affecting the lytic R transactivator and the A6 gene may be associated with increased fitness in culture (7) (GenBank accession no. KX905136, WC11 duplicated region; KX905135, C500 bacmid virus; KX905134, C500 bacmid). Coverage analysis of strain WC11 supports neither the presence of this duplication nor its relocation to the terminal repeat of H-DNA. In WC11, however, the neighboring Z transactivator A6 and glycoproteins A7 and A8 are affected by the deletion at positions 76206 to 78373. ORF A6 is joined to ORF A8, replacing the A6 carboxy terminus of 10 amino acids (aa) with 15 aa in an alternative reading frame within A8. Moreover, 245 single and multiple nucleotide variants are present in the WC11 127-kb L-DNA genome, indicating that, overall, it is 99.8% identical to the C500 genome; notably, most of the variation is accounted for by a variable region (98% identity) between positions 124000 and 129500. Genomic rearrangements and the deletions of ORF A1, miR cluster 1, and two glycoproteins (A7 and A8) as described here may have resulted in the attenuation of strain WC11.

### Accession number(s).

This whole-genome sequence of strain WC11 has been deposited at GenBank under the accession no. MG000864.
